# Rotavirus-Induced Lipid Droplet Biogenesis Is Critical for Virus Replication

**DOI:** 10.3389/fphys.2022.836870

**Published:** 2022-03-23

**Authors:** Jeanette M. Criglar, Mary K. Estes, Sue E. Crawford

**Affiliations:** ^1^ Department of Molecular Virology and Microbiology, Baylor College of Medicine, Houston, TX, United States; ^2^ Department of Medicine, Baylor College of Medicine, Houston, TX, United States

**Keywords:** lipid droplets, rotavirus, viroplasm, fatty acid synthesis, pathogen

## Abstract

A variety of pathogens, including viruses, bacteria and parasites, target cellular lipid droplets for their replication. Rotaviruses (RVs) infect the villous epithelium of the small intestine and are a major cause of acute gastroenteritis in infants and young children worldwide. RVs induce and require lipid droplets for the formation of viroplasms, sites of virus genome replication, and nascent particle assembly. Here we review the role of lipid droplets in RV replication. Inhibitors of fatty acid synthesis or chemicals that interfere with lipid droplet homeostasis decrease the number and size of viroplasms and the yield of infectious virus. We used a genetically engineered RV, delayed in viroplasm assembly, to show an early interaction of RV nonstructural protein NSP2 and the lipid droplet-associated protein phospho-PLIN1. The interaction between NSP2 and phospho-PLIN1 suggests that we have identified part of the mechanism of RV-induced lipid droplet formation. These studies demonstrate that RV is an excellent model to dissect the cellular process of lipid droplet formation and to determine how RV induces and usurps lipid droplet biogenesis to form viroplasm/lipid droplets for virus replication.

## Introduction

Rotaviruses (RVs) infect intestinal enterocytes, enteroendocrine and tuft cells in the small intestine causing vomiting and diarrhea that can be life-threatening due to severe dehydration in infants and young children (Hagbom et al., 2011; Crawford et al., 2017; Bomidi et al., 2021). Prior to the introduction of RV vaccines in 2006, RV caused 500,000 deaths annually worldwide; yet, despite the positive impact of the vaccines, RV infection still results in an estimated 128,000 deaths globally and highlights the importance for the continued study of this significant pathogen (Troeger et al., 2018).

RVs are nonenveloped particles that have a complex architecture consisting of three concentric capsid layers surrounding a genome of 11 double-stranded RNA (dsRNA) segments encoding six structural proteins (VP1/2/3/4/6/7) that form the virus particle, and six nonstructural proteins (NSP1-6; NSP6 is only present in certain virus strains) that play various roles in RV replication. RV dsRNA replication and immature double-layered particle (DLP) assembly occur in electron-dense structures, known as viroplasms, that form in the cytoplasm of infected cells. Viroplasms are composed of both viral and cellular proteins, and in 2010 components of lipid droplets were found associated with viroplasms, potentially linking viroplasm formation and lipid droplet biogenesis (Cheung et al., 2010).

Lipid droplets were first described in 1886 by Edmund Wilson as “oil-drops” in developing sea urchin eggs, and until relatively recently, were considered cellular organelles with the sole purpose of storing “fat”. Lipid droplets are now recognized as playing diverse roles in cellular lipid metabolism, energy metabolism and cell signaling (Thiam et al., 2013), and are implicated in multiple human infectious diseases including hepatitis, chlamydia, tuberculosis, and metabolic diseases such as diabetes and atherosclerosis. Lipid droplets are spherical structures with a neutral lipid core containing triacylglycerol (Tauchi-Sato et al., 2002) and sterol esters. The lipid droplet core is surrounded by a phospholipid monolayer that is decorated with various cellular proteins, in particular, the lipid droplet-associated proteins, or perilipins (PLIN 1-5). However, the mechanism by which lipid droplets form remains poorly understood (Walther et al., 2017; Olzmann and Carvalho, 2019; Thiam and Ikonen, 2021).

Several RV nonstructural (NSP2/5/6) and structural (VP1/2/3/6) proteins, as well as components of LDs, are detected in viroplasms. Viroplasm assembly requires NSP2 and NSP5; inhibition of either protein prevents viroplasm formation and profoundly reduces the yield of infectious virus (Silvestri et al., 2004; Vascotto et al., 2004; Campagna et al., 2005; Papa et al., 2019). The mechanism of viroplasm assembly and the association with lipid droplet remain largely unknown. Geiger et al. suggest that viroplasm assembly occurs by liquid-liquid phase separation in which the RNA-binding protein NSP2 and the intrinsically disordered NSP5 protein form protein-RNA condensates (Geiger et al., 2021). We previously discovered that the NSP2 protein exists in two structurally distinct forms: as a cytoplasmically dispersed form (dNSP2) and a viroplasm-localized form (vNSP2) that are distinguished by conformation-specific monoclonal antibodies (Criglar et al., 2014). Notably, dNSP2 and vNSP2 interact differentially with hypo- and hyper-phosphorylated NSP5, respectively, and this led to the discovery of a coordinated phosphorylation-dependent mechanism that regulates viroplasm assembly (Criglar et al., 2018); phosphorylation of dNSP2 on serine 313 by the cellular kinase CK1α triggers the localization of vNSP2 (formed by an as yet unidentified mechanism) to sites of viroplasm assembly and its association with hyperphosphorylated NSP5 in viroplasms (Criglar et al., 2020). However, a fundamental question remains: Do either NSP2 or NSP5 physically interact with lipid droplet components to trigger lipid droplet biogenesis? The goal of this review is to examine studies that support the hypothesis that rotavirus infection induces and requires lipid droplets for replication.

### Rotavirus Infection Induces the Formation of Viroplasm/Lipid Droplets

**FIGURE 1:**
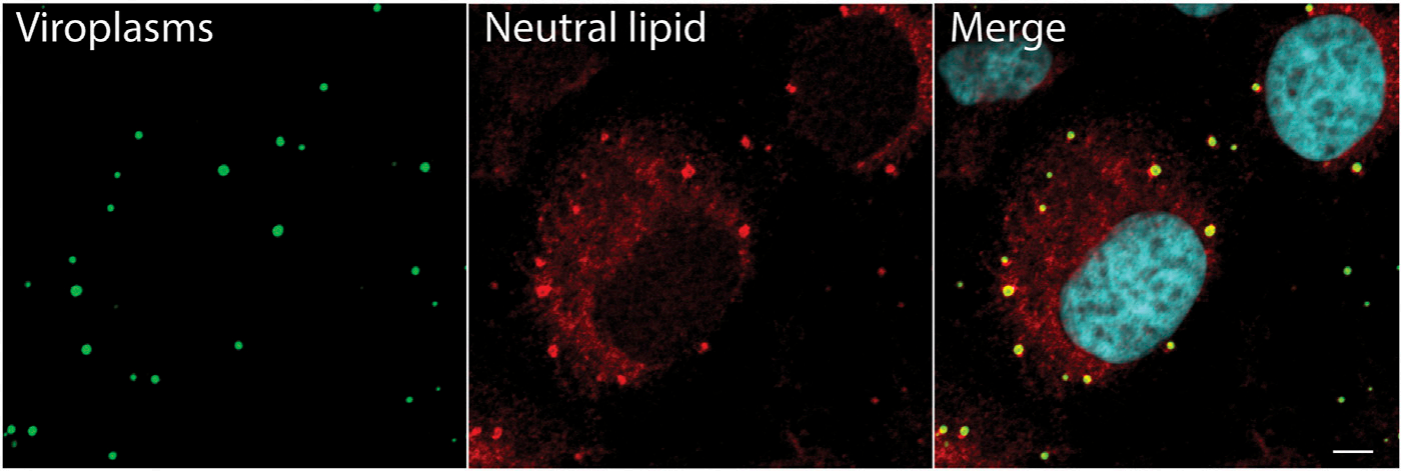
Neutral lipid containing lipid droplets colocalize with viroplasms. Immunofluorescence confocal microscopy was used to detect viroplasms (anti-NSP2, green) and neutral lipid located inside of lipid droplets (LipidTox, red) in RV-infected cells 4 hpi (MOI 5). An uninfected cell is seen at the top of the image, on the left. Nuclei were detected with DAPI (light blue). Scale bar = 5 µm. Previously unpublished data. Immunofluorescent assay performed as described in Criglar, 2020 (Criglar et al., 2020).

The first evidence that RV viroplasms associate with components of lipid droplets came from immunofluorescence microscopy studies that showed that both RV viroplasm-associated proteins NSP2 and NSP5 co-localize with lipid droplet-associated proteins PLIN1 and PLIN2 (Cheung et al., 2010). It was also shown that viroplasms colocalize with the lipophilic stain Nile Red, further suggesting colocalization of viroplasms with lipid droplets (Cheung et al., 2010). However, one drawback of using Nile Red for these studies is that Nile Red non-specifically labels all cellular lipid organelles and intracellular membranes. Therefore, to confirm these results, we used LipidTOX™, a neutral lipid stain that has an extremely high affinity for neutral lipids to detect lipid droplets and guinea pig anti-NSP2 to detect viroplasms. Using immunofluorescence and confocal microscopy, we showed that the majority of lipid droplets detected with LipidTOX™ colocalized with the NSP2 in viroplasms (Figure 1). Lipid droplets detected with LipidTOX™ are not observed in neighboring cells (Figure 1, top left cell). This result confirms the earlier report that viroplasms colocalize with lipid droplets.

**FIGURE 2:**
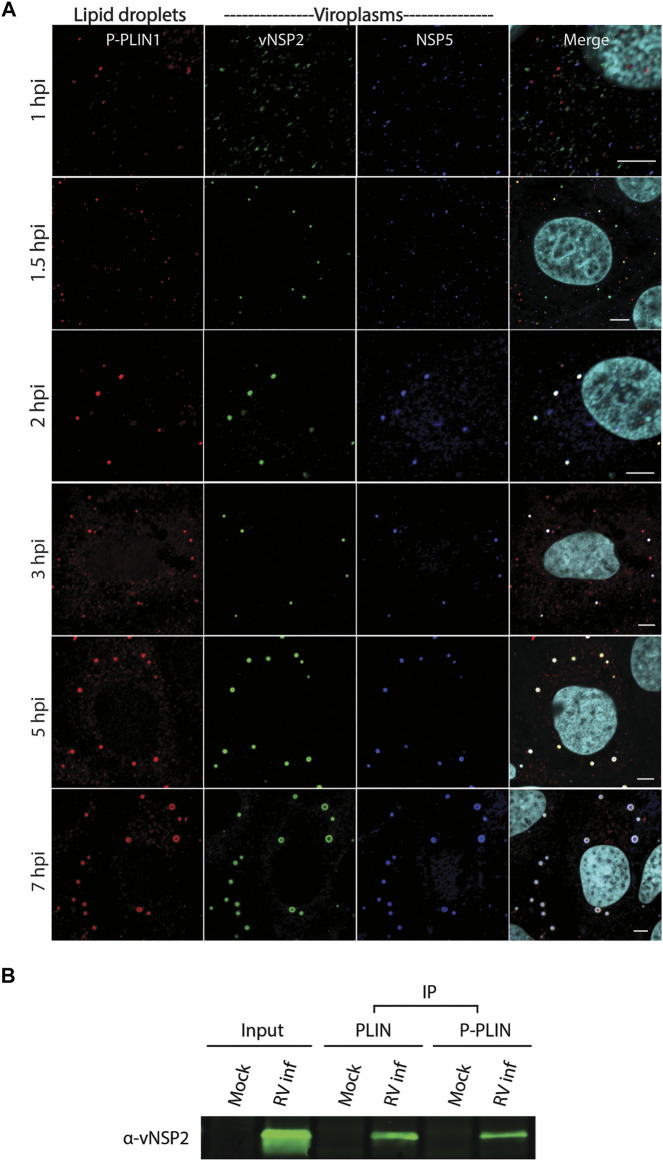
RV viroplasmic proteins vNSP2 and NSP5 colocalize with lipid droplets early in infection. **(A)** Immunofluorescence confocal microscopy was used to detect lipid droplets and viroplasms in RV-infected cells at 1, 1.5, 2, 3, 5, and 7 hpi. Mouse anti-phospho-PLIN1 (red) was used to detect lipid droplets. Viroplasms were detected using mouse monoclonal antibody anti-vNSP2 (green), and guinea pig polyclonal antibody anti-NSP5 (blue). Nuclei were detected with DAPI (light blue). Scale bar = 5 µm. Previously unpublished data. Immunofluorescent assay performed as described in Criglar, 2020 (Criglar et al., 2020). **(B)** Western blot of vNSP2 co-immunoprecipitated with PLIN1 or P-PLIN1 from RV-infected cell lysates. [From Criglar, et al., 2020 (Criglar et al., 2020)].

To determine whether viroplasms form prior to, or concomitantly, with lipid droplets we performed a time-course experiment. Viroplasms and lipid droplets were imaged in RV-infected cells at 1, 1.5, 2, 3, 5, and 7 h post infection (hpi). We probed RV-infected cells for the lipid droplet-specific phosphorylated PLIN1 (P-PLIN1) that is only found on lipid droplets. Viroplasms were detected by the monoclonal antibody that detects viroplasmic NSP2 (vNSP2) and a polyclonal antibody anti-NSP5 (Figure 2A). At 1 hpi, small puncta of vNSP2, P-PLIN1 and NSP5 are detected in RV-infected cells. Most of these puncta do not colocalize. However, as early as 1.5 hpi, vNSP2, and NSP5 colocalize with P-PLIN1. As infection progresses, the size of both the viroplasms and lipid droplets increase (Eichwald et al., 2004), suggesting that viroplasms assemble concomitantly with lipid droplet biogenesis. It should be noted that detection of NSP2 colocalized with NSP5 very early in infection does not prove that the observed puncta are replication-competent viroplasms; they could be NSP2/NSP5 complexes that form prior to viroplasm assembly. Determining the precise temporal and serial steps leading to viroplasm/lipid droplet formation will require detailed analysis using high resolution microscopy, reverse genetics generated viruses mutated in ability to form viroplasms, and detection of other viroplasm and lipid droplet proteins and neutral lipid.

The growth of viroplasm/lipid droplets during the virus replication cycle may include recruitment of other lipid droplet-specific proteins and increased neutral lipid in the lipid droplet core. To determine if there was a direct interaction between vNSP2 and PLIN1, PLIN1 and P-PLIN1 were immunoprecipitated using monoclonal antibodies to the unmodified or phosphorylated forms of perilipin from RV-infected cell lysates. Western blot analysis showed that vNSP2 was coimmunoprecipitated with both PLIN1 and P-PLIN1, suggesting a direct interaction between NSP2 and both forms of PLIN1 (Figure 2B) (Criglar et al., 2020).

### Inhibition or Disruption of Lipid Droplets Reduces Viroplasm Formation and Virus Replication

**FIGURE 3:**
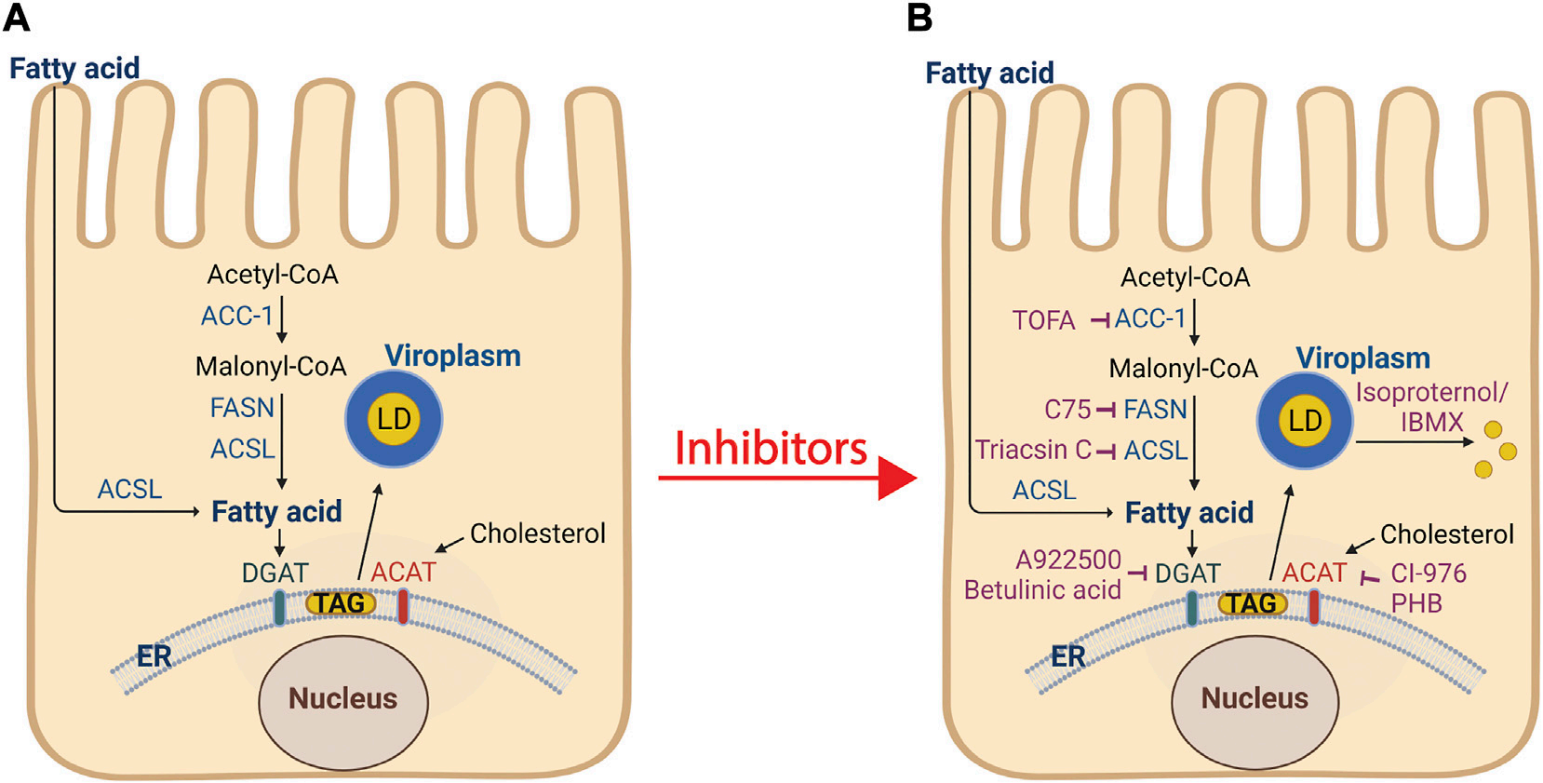
Processes of lipogenesis and lipid droplet biogenesis, and interventions that disrupt viroplasm formation. **(A)** De novo fatty acid synthesis involves the conversion of acetyl-CoA into malonyl-CoA by acetyl-CoA carboxylase 1 (ACC-1). Fatty acid synthase (FASN) catalyzes the synthesis of the fatty acid palmitate from acetyl-CoA and malonyl-CoA. Long chain acyl-CoA synthetase (ACSL) facilitates extracellular fatty acid uptake and converts fatty acids into their corresponding CoA esters for oxidation or esterification into complex lipids (e.g., triglycerides, phospholipids, and cholesterol esters). The ER-localized enzymes diacylglycerol acyltransferases (DGAT1 and DGAT2), and acyl-coenzyme A (CoA): cholesterol acyltransferases (ACAT1 and ACAT2) synthesize triacylglycerol (Tauchi-Sato et al., 2002) from fatty acids and sterol esters from cholesterol, respectively. These products are stored in the lipid bilayer of the ER. The current model of lipid droplet biogenesis posits that the TAG and sterol esters accumulate and by an unknown mechanism bud from the ER into the cytoplasm and acquire lipid droplet-associated proteins (PLINs 1-5). RV viroplasms associate with lipid droplets. **(B)** Inhibitors (shown in purple) that block lipid droplet formation or disperse lipid droplets significantly decrease the number and size of viroplasms and the amount of infectious viral progeny. Treatment of cells with isoproterenol and IBMX fragment lipid droplets into smaller microdroplets. Image created with BioRender (BioRender.com). Modified from Crawford and Desselberger, 2016 (Crawford and Desselberger, 2016).

Cellular lipogenesis requires free fatty acid (provided to the intestine by diet or via *de novo* synthesis by the reiterative catalysis of acetyl-CoA with malonyl-CoA) and glycerol to form triglycerides (TAGs) (Tauchi-Sato et al., 2002). TAG contains a backbone of glycerol and three fatty acid molecules and are the primary storage molecule for energy in the body. Numerous enzymes participate in lipogenesis, including acetyl-CoA carboxylase (ACC-1), fatty acid synthase (FASN), long chain fatty acid acyl-CoA synthetase (ACSL) 1-6, and diacylglycerol acyltransferase (DGAT) 1 and 2 (Figure 3A). DGAT catalyzes the formation of TAG from diacylglycerol and free fatty acids, and newly synthesized TAG is stored in the lipid bilayer of the endoplasmic reticulum (ER). In addition to TAG, sterol esters are synthesized from cholesterol by cholesterol acyltransferase (ACAT) 1 and 2 and are also stored in the ER lipid bilayer. While the mechanistic details of lipid droplet biogenesis remain poorly understood, the current model posits that TAG and sterol esters accumulate between the leaflets of the ER lipid bilayer and a TAG/sterol ester lipid droplet is released into the cytoplasm.

The complex formation of viroplasm/lipid droplets are essential for RV replication as knockdown of NSP2 or NSP5 proteins, or disruption of lipolysis, significantly decrease the number and size of viroplasms and the amount of infectious progeny produced (Cheung et al., 2010). Silencing the expression of NSP2 or NSP5 by RNA interference (Silvestri et al., 2004; López et al., 2005), or intrabodies (Vascotto et al., 2004), or RV infections with a NSP2 *temperature-sensitive* (*ts*) mutant at the non-permissive temperature (Taraporewala et al., 2002), prevent viroplasm formation and virion production. Interruption of lipogenesis and/or lipid droplet biogenesis pathways at any number of steps disrupts RV viroplasm formation and replication (Figure 3B). Treatment of RV-infected cells with the ACC-1 inhibitor TOFA, 4 h *prior* to infection, showed the greatest reduction in viral and dsRNA production, although a significant decrease in both these factors was still observed when TOFA was added at 4 h *post* infection (Gaunt et al., 2013a). The FASN inhibitor C75 showed a modest effect on virus production, but in combination with TOFA, a synergistic reduction in viral yield was reported (Gaunt et al., 2013a). Treatment of RV-infected cells with the ACSL inhibitor triacsin C reduced viroplasm size and number with a corresponding reduction in viral yield (Cheung et al., 2010; Kim et al., 2012). Inhibitors of DGAT (A922500 or betulinic acid) or ACAT (CI-976 or PHB) also reduced viral yields (Kim et al., 2012). Dispersion of lipid droplets into smaller microdroplets with isoproterenol and IBMX resulted in reduced number and size of viroplasms, decreased production of viral dsRNA, and a 120–200-fold lower yield of infectious progeny (Cheung et al., 2010). In addition, Cheung, et al. found the viability of the isoproterenol and IBMX-treated, RV-infected cells was significantly higher at later time points post infection as compared to non-treated RV-infected cells, suggesting that the reduction in RV-induced cytopathicity is correlated with increased cell viability (Cheung et al., 2010).

### Lipidome Analyses Show the Total Lipid Content of RV-Infected Cells Is Significantly Higher When Compared to Uninfected Cells

In mammalian cells, the lipid droplet surface is composed of a phospholipid monolayer. The most abundant phospholipid in the monolayer is phosphatidylcholine (PC), followed by phosphatidylethanolamine (PE), phosphatidylinositol, lysoPE, and lysoPC, but the membrane is deficient in sphingomyelin (SM), phosphatidylserine (PS), and phosphatidic acid (PA) (Tauchi-Sato et al., 2002; Bartz et al., 2007). Comparative analysis of the lipidome of mock- and RV-infected cells was performed to gain insight into whether the phospholipid composition of the monolayer was altered in RV-infected cells. Lipidome analysis was performed on iodixanol gradient fractions that co-sedimented with RV dsRNA genome and lipid droplet- and viroplasm-associated proteins. The results demonstrated that the total cellular lipid content, including phospholipids found in lipid droplets, increases during RV infection compared to mock-infected cells (Kim and Chang, 2011; Gaunt et al., 2013b) and that the lipid increase is consistent with an increase in abundance of lipid droplets (TAG, sterol esters) that interact with viroplasms (Gaunt et al., 2013b). Phospholipids such as ceramide, PS, PA, and SM were also increased.

The increase in other, non-lipid droplet associated phospholipids in RV-infected cells may be explained by the increase of other membranes that associate with viroplasms. In a morphogenic step that is exceedingly rare for nonenveloped viruses, RV virion intermediate, double-layered particles (DLPs), form in and emerge from the periphery of the viroplasm and bud through viroplasm-associated cytoplasmic cellular membranes to acquire the outer capsid proteins, VP4 and VP7, resulting in mature, infectious particle assembly. This morphogenic step is mediated by the RV nonstructural protein NSP4. NSP4, synthesized as an ER transmembrane glycoprotein, is a viroporin that releases calcium from the ER store into the cytoplasm that in turn activates the cellular process of autophagy (Crawford et al., 2012; Crawford and Estes, 2013). Autophagy is an intracellular membrane trafficking pathway and a lysosome-mediated degradation process by which cells digest their own damaged organelles and macromolecules to meet bioenergetic needs and enable protein synthesis. NSP4 exits the ER in COPII vesicles that are hijacked by the autophagy process by the direct interaction of NSP4 with the autophagy protein LC3 II (Crawford et al., 2019). The NSP4/LC3 II-containing membranes accumulate adjacent to viroplasms (Crawford et al., 2019). Other studies demonstrated that rotavirus induces cellular autophagy but LC3 was not detected adjacent to viroplasms (Arnoldi et al., 2014). This may be due to the antibody or conditions used to detect LC3 in membranes by confocal microscopy. The C-terminal cytoplasmic domain of NSP4, amino acids 161–175, binds the inner coat protein (VP6) of DLPs in viroplasms (Au et al., 1989; Meyer et al., 1989; O'Brien et al., 2000; Taylor et al., 1996). This interaction triggers the budding of the DLP through the NSP4-containing membranes where the particles become transiently enveloped. The transient lipid envelope is removed by an unknown mechanism and the outer capsid proteins, VP7 that traffics with NSP4 to viroplasms and VP4 that interacts with NSP4, are assembled onto the DLPs to form mature, infectious triple-layered particles (TLPs).

Many RNA viruses dramatically remodel and accumulate host cellular membranes required for viral replication (Netherton et al., 2007; Miller and Krijnse-Locker, 2008; de Armas-Rillo et al., 2016). The increase in phospholipids not associated with lipid droplets detected from the RV-infected cells may be due to the increase in NSP4/LC3 II-containing membranes that are in close association with viroplasms. The mechanisms by which NSP4/LC3 II-containing membranes and lipid droplet membranes accumulate and obtain a continuous supply of phospholipid remain to be fully elucidated. It is possible that the neutral lipids stored in the lipid droplets are mobilized for phospholipid synthesis during high demand for membranes (Olzmann and Carvalho, 2019). This is an intriguing possibility because TOFA treatment of RV-infected cells not only caused a 2-fold reduction in the production of RV DLPs, but a 20-fold reduction in detectable TLPs (Gaunt et al., 2013a; Cheung et al., 2016). This result suggests inhibition of fatty acid synthesis may affect RV replication not only at the steps occurring within viroplasms up to DLP formation but also at the later steps of infectious virus assembly (TLPs).

### Viroplasm/Lipid Droplets Form in Human Intestine Stem Cell-Derived Enteroids

**FIGURE 4:**
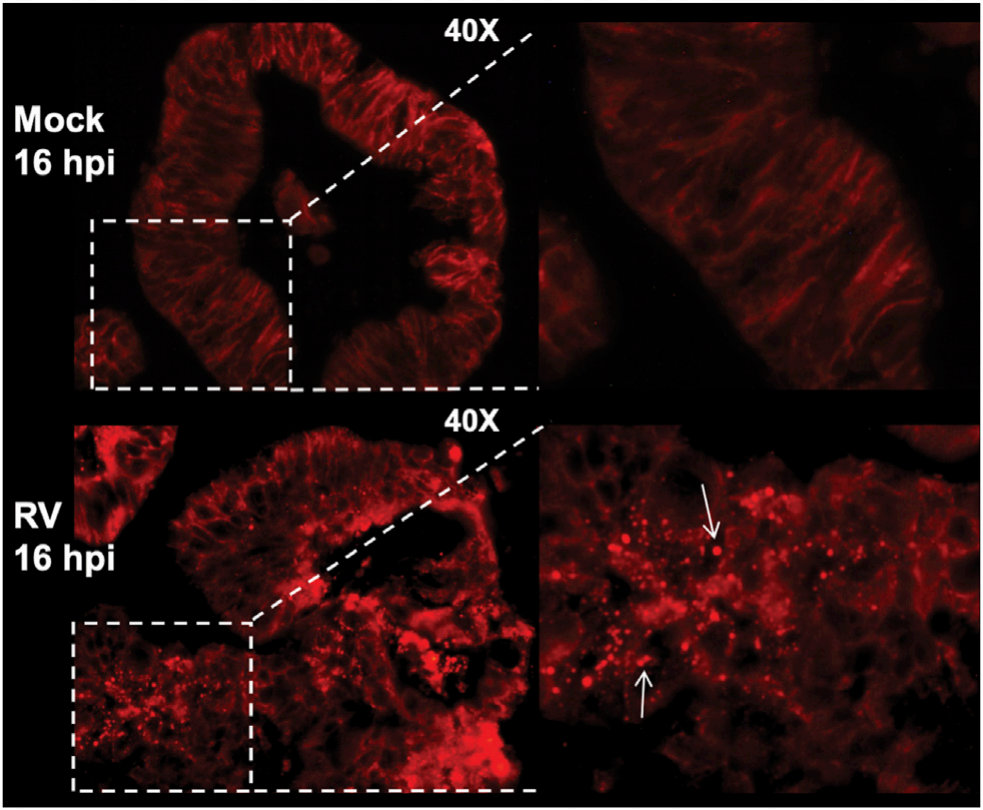
RV-infection of human intestinal enteroids induces lipid droplets. Human jejunal enteroids were metabolically labeled with fluorescently-tagged BODIPY-fatty acid for 1 h prior to mock or RV infection. Enteroids imaged by epifluorescence at 16 hpi show BODIPY-fatty acid is retained in the endoplasmic reticulum lipid bilayer of mock-infected enteroids (top panel). In contrast, RV infection induces lipid droplet formation indicated by the round, red lipid droplets (bottom panel, arrows). The inset on the right is a magnification of the boxed area. [From Foulke-Abel et al., 2014 (Foulke-Abel et al., 2014)].

**FIGURE 5:**
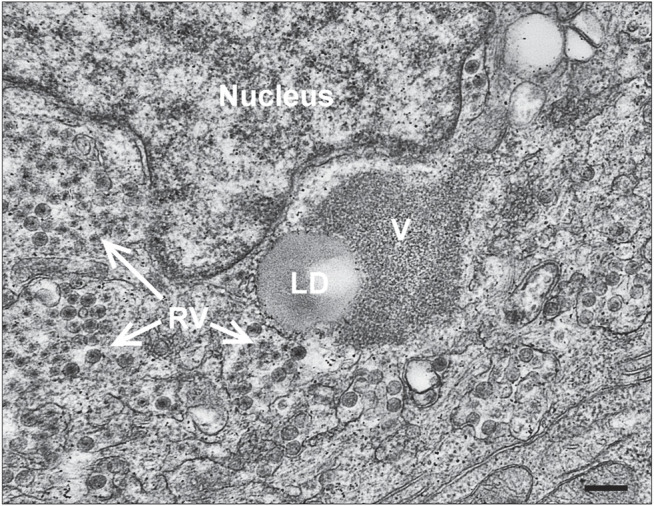
Viroplasm/lipid droplets form in RV-infected HIEs. Electron micrograph of an infected cell within an enteroid. RV particles adjacent to a lipid droplet (LD) and viroplasm (V) are shown (bar = 250 nm). [From Saxena et al., 2016 (Saxena et al., 2016)].

Much has been learned of human host–enteric pathogen interactions from studies using cancer cells, animal models, clinical data, and occasionally, controlled human infection models. However, in-depth interrogation of the complex interactions between human viruses and the human intestinal epithelium are now possible with the advent of human intestine stem cell-derived enteroids (HIEs). These nontransformed, multicellular, physiologically active, and organotypic cultures recapitulate the relevant heterogenous cell types that comprise the intestinal villus epithelium. Recent studies using HIEs are revealing previously unknown host-microbe interactions that affect pathogen replication and outcomes of human infections (Crawford et al., 2021). Infection of HIEs with human RVs have demonstrated host range and cell type restriction, and virus-induced fluid secretion (Saxena et al., 2016). Intracellular changes typical of RV replication in cultured cells are seen in infected HIEs and include the induction of lipid droplets (Figure 4) (Foulke-Abel et al., 2014). Transmission electron microscopy of human RV-infected HIEs show a viroplasm/lipid droplet and large numbers of triple-layered particles as well as budding triple-layered particles (Figure 5) (Saxena et al., 2016). These studies demonstrate RV-infected HIEs are an excellent, biologically relevant, model system to investigate complex cellular mechanisms of lipid droplet biogenesis.

### A Genetically Engineered RV Reveals an Early Interaction Between NSP2 and Lipid Droplets

**FIGURE 6:**
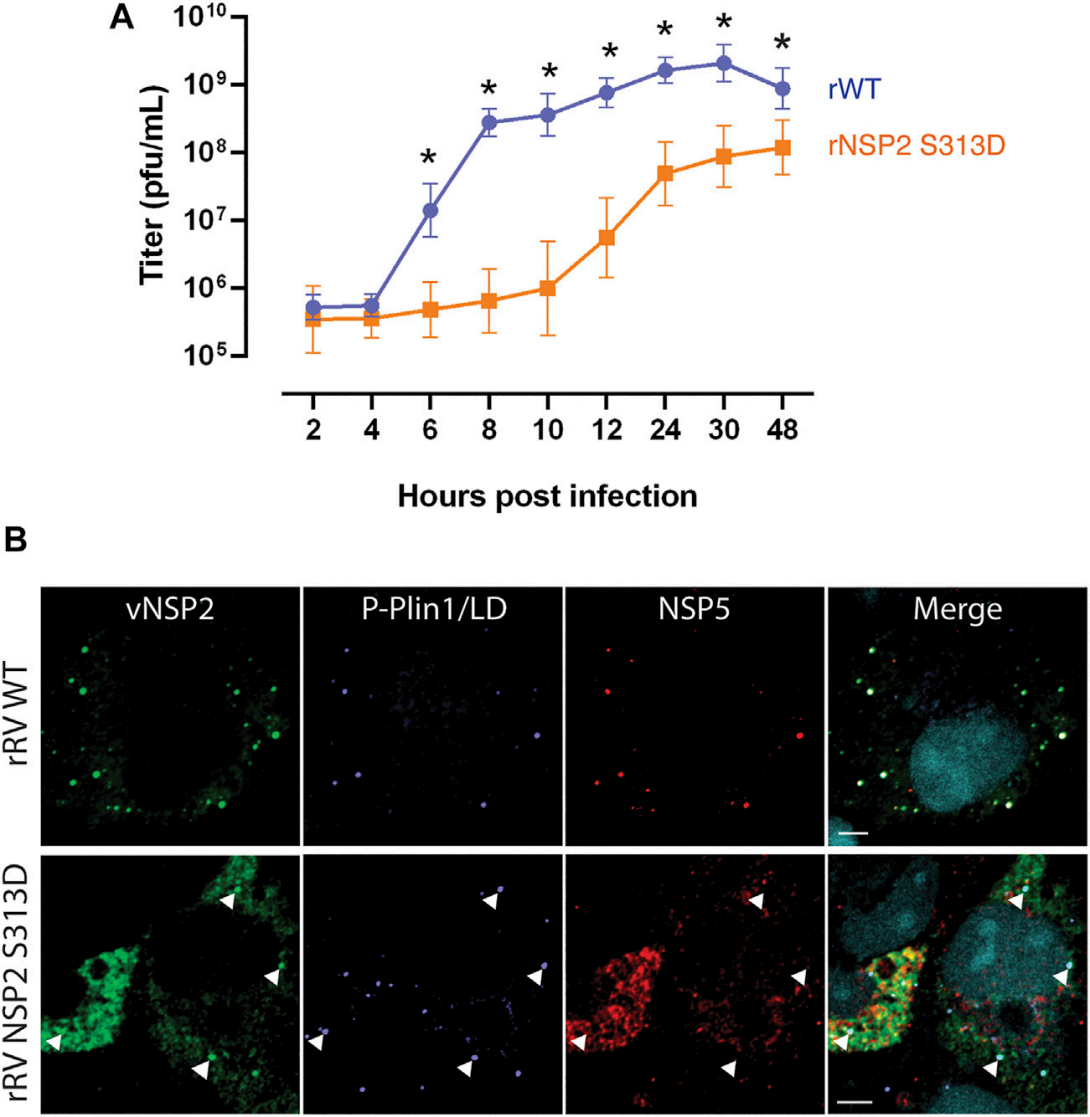
vNSP2 expressed by the RV NSP2 S313D phospho-mimetic mutant virus colocalizes with nascent lipid droplets before association with NSP5. **(A)** Growth curves of recombinant RV (rRV) wild type compared to phosphomutant rRV NSP2 S313D virus showing the delay in virus replication as a consequence of delayed viroplasm formation. **(B)** Representative images of MA104 cells infected with rRV WT (top panel) or rRV NSP2 S313D (bottom panel) virus at 4 hpi. vNSP2, P-PLIN1, and NSP5 colocalize in rRV WT-infected cells; whereas, in rRV NSP2 S313D virus-infected cells vNSP2 colocalizes with RV-induced P-PLIN1 decorated lipid droplets before interacting with NSP5 (examples are indicated with white arrowheads). Scale bar = 5 µm vNSP2 was detected with mouse monoclonal antibody anti-vNSP2 (green). Mouse anti-phospho-PLIN1 (blue) was used to detect lipid droplets. NSP5 detected with guinea pig polyclonal antibody anti-NSP5 (red). Nuclei were detected with DAPI (light blue). Scale bar = 5 μm [From Criglar et al., 2020 (Criglar et al., 2020)].

We previously reported that phosphorylation of NSP2 on serine 313 by the cellular kinase CK1α triggers the localization of vNSP2 to sites of viroplasm assembly and its association with hyperphosphorylated NSP5 (Criglar et al., 2018). To directly evaluate the role of CK1α-mediated NSP2 phosphorylation on viroplasm formation, we generated a recombinant RV (rRV) with a phosphomimetic NSP2 mutation (rRV NSP2 S313D). The rRV NSP2 S313D virus is significantly delayed in viroplasm formation, virus replication (Figure 6A), and interferes with wild type RV replication during co-infection (Criglar et al., 2020). Taking advantage of the delay in viroplasm formation, the NSP2 S313D phosphomimetic mutant was used as a tool to observe very early events in viroplasm assembly. At 4 hpi in wild type RV-infected cells, both NSP2 and NSP5 are detected colocalized with P-PLIN1 in nascent viroplasms (Figure 6B, upper panel). The unphosphorylated form of PLIN1 inhibits lipolysis, the metabolic process by which triglycerides are hydrolyzed into glycerol and fatty acids; whereas, PLIN1 phosphorylation leads to lipolysis (Tansey et al., 2004). Because viroplasm formation occurs too rapidly in wild type virus infection to accurately determine if there is an order to the assembly of the NSP2/NSP5/P-PLIN1 complex, we examined cells infected with the rRV NSP2 S313D mutant at the same 4-h time point post infection (Figure 6B, lower panel). We determined that NSP2 D313 associates with P-PLIN1 prior to interaction with NSP5, as no P-PLIN1/NSP5 colocalization was observed without vNSP2, suggesting that vNSP2 phospho-S313 is sufficient for interacting with lipid droplets and may be the virus factor required for RV-induced lipid droplet formation. This result substantiates our observation that viroplasms assemble concomitantly with lipid droplets. Further studies with the rRV NSP2 S313D virus are expected to reveal new aspects of viroplasm and LD initiation and assembly.

## Concluding Remarks and Future Directions

Viruses exploit host cells by remodeling membranes, subverting host proteins and co-opting cellular metabolic pathways to create a cellular environment conducive to infection, replication, evasion of host defense and facilitate viral egress. Here we review how RV exploits lipid droplet metabolism during the RV replication cycle. Lipid droplets are now recognized as crucial organelles for the replication of many pathogens: members of the *Flaviviridae* family (Miyanari et al., 2007; Samsa et al., 2009; Chatel-Chaix and Bartenschlager, 2014) and SARS-CoV-2 (Dias et al., 2020), the parasite *Trypanosoma cruzi* (Melo et al., 2003), and intracellular bacteria *Chlamydia* (Kumar et al., 2006) and *Mycobacterium tuberculosis* (Daniel et al., 2011).

Clinically, we do not understand why RV-induced gastroenteritis causes more severe, life-threatening disease compared to other enteric infections. In addition, children in poor environmental conditions who develop repeat infections might sustain long-term damage to the gut, which can lead to consequences on physical, and cognitive development (Kolling et al., 2012; Crawford et al., 2017). Considering RV infects and replicates in the mature, non-dividing enterocytes in the middle and tip of the villi, the major site of fat absorption in the body, and in enteroendocrine cells in the small intestine (Hagbom et al., 2011; Crawford et al., 2017), it is not surprising that RV would exploit lipogenesis pathways and lipid droplets as structural platforms for virus replication. Experimentation with HIOs as a RV replication system that more closely mimics the human intestinal epithelium may address the intriguing questions that remain (Table 1).

**TABLE 1:** Current questions for pathogen-induced lipid droplet biogenesis research.

Do pathogens use common mechanisms to induce lipid droplets?
Which cellular and pathogen proteins are required for pathogen-induced lipid droplet biogenesis?
Can cellular proteins or mechanisms common in pathogen-induced lipid droplet biogenesis be targeted to provide pan-antimicrobial therapeutics?
Does lipid droplet lipolysis play a role in pathogen replication or disease?
How do rotaviruses induce lipid droplet biogenesis?
Do rotaviruses induce lipid droplet biogenesis simply as a replication platform or do lipid droplets play other roles in rotavirus replication and pathogenesis?
What is the role of the PLIN proteins in rotavirus-induced lipid droplet biogenesis?
Which rotavirus proteins interact with lipid droplets and how?
